# Advancements in Catalyst Design for Biomass‐Derived Bio‐Oil Upgrading to Sustainable Biojet Fuel: A Comprehensive Review

**DOI:** 10.1002/open.202500353

**Published:** 2025-09-14

**Authors:** Thandiswa Jideani, Ntalane Sello Seroka, Lindiwe Khotseng

**Affiliations:** ^1^ Department of Chemistry, Faculty of Natural Sciences University of the Western Cape Robert Sobukwe Road, Private Bag X17 Bellville 7535 South Africa; ^2^ Energy Centre, Smart Places Cluster Council for Scientific and Industrial Research (CSIR) Pretoria 0001 South Africa

**Keywords:** bio‐oil, biomass, catalysts, hydrothermal liquefaction, pyrolysis

## Abstract

Biomass‐derived bio‐oil, produced through thermochemical methods such as pyrolysis and hydrothermal liquefaction, has immense potential as a renewable feedstock for aviation fuels because of its renewable nature and the potential to significantly reduce greenhouse gas emissions. The development of biojet fuel from renewable sources, such as biomass, is a critical step toward achieving global energy sustainability and reducing the carbon footprint of the aviation industry. This review aims to provide a comprehensive analysis of the advances in catalyst design to upgrade biomass‐derived oil to biojet fuel. The review will also explore the mechanisms by which these catalysts operate, the optimization of catalytic processes, and the performance metrics used to evaluate their efficiency. Recent case studies demonstrate the effectiveness of catalyst design in enabling efficient and sustainable conversion of biomass‐based bio‐oil into high‐quality fuels, advancing the viability of renewable energy sources in aviation and beyond.

## Graphical abstract

The design of hierarchical ZSM‐5 catalysts for the conversion of biomass‐derived bio‐oil, specifically from sugarcane bagasse, into biojet fuel. The underlying mechanisms underscore the significance of producing biojet fuel from renewable resources to boost global energy sustainability and lower carbon emissions within the aviation industry.

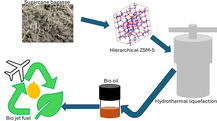

## 1 Introduction

### 1.1 Background on Biomass‐Derived Bio‐Oil

The increasing global demand for sustainable energy solutions has intensified research on renewable fuel sources, with biomass‐derived bio‐oil emerging as a promising candidate.^[^ ^1^ ^]^ Growing concerns about climate change, depletion of fossil fuel resources, and the environmental impact of conventional petroleum‐based fuels have driven the search for alternatives. In response, the use of biomass, a readily available and renewable resource, has gained significant attention for the production of cleaner fuels.^[^ ^2^ ^]^

Biomass, which includes a wide range of organic materials, such as agricultural residues, forest biomass, and dedicated energy crops, is subjected to thermochemical processes such as hydrothermal liquefaction (HTL) or pyrolysis to produce bio‐oil.^[^ ^3^ ^]^ This resulting bio‐oil is a dark, viscous, and complex mixture of oxygenated hydrocarbons and presents a viable alternative to fossil fuels because of its renewable nature and the potential to significantly reduce greenhouse gas emissions. However, the direct use of bio‐oil is limited by its high oxygen content, acidity, and instability, which require further upgrading to convert it into higher‐quality fuels.^[^ ^4^ ^]^

Although biomass bio‐oil production presents a viable renewable energy source, its direct use as a fuel is limited by several factors. Bio‐oil typically contains a high concentration of oxygenated compounds, which can comprise up to 40% of its total weight. This high oxygen content results in undesirable properties such as high acidity, instability, low heating value, and high water content.^[^ ^5^ ^]^ Carboxylic acids make bio‐oil highly acidic, with a pH ranging between 2 and 4, leading to corrosion issues in engines and storage tanks. Furthermore, bio‐oil is thermally and chemically unstable, undergoing polymerization and phase separation over time, which reduces its shelf life and increases its viscosity.^[^ ^6^ ^]^

Its high oxygen content also reduces its energy density compared to conventional hydrocarbon fuels, and its water content, which can be as high as 30%, further reduces its heating value and affects combustion performance.

The earliest catalytic strategies for bio‐oil upgrading were adapted directly from petroleum hydroprocessing. Sulfided CoMo and NiMo on *γ*‐Al_2_O_3_ longstanding workhorses for diesel hydrotreating were applied to model oxygenates and then to real fast‐pyrolysis oils for hydrodeoxygenation (HDO).^[^ ^7^ ^]^ These catalysts established the feasibility of deep oxygen removal to aviation‐range hydrocarbons but also exposed biomass‐specific issues: severe coking driven by reactive phenolics and aldehydes, rapid deactivation under high water partial pressures, and the need for sulfur make‐up to maintain active sulfide phases in ultra‐low‐sulfur bio‐feeds. Subsequent waves of work explored supports beyond alumina ZrO_2_, TiO_2_, SiO_2_ ZrO_2_, carbon—to improve hydrothermal tolerance and tune metal–support interactions that govern C–O bond scission versus ring‐saturation selectivity.^[^ ^8^ ^]^ In parallel, noble metals (Pd, Pt, Ru) on carbon and oxides demonstrated high HDO activity at lower temperatures and without sulfur, but their cost and susceptibility to poisoning by N/Cl species motivated development of bimetallic and promoted base‐metal systems (e.g., Ni–Mo, Ni–W, Ni–Cu, P‐ or B‐modified formulations) with improved dispersion, hydrogenation function, and coking resistance.^[^ ^9^ ^,^ ^10^ ^]^

On the cracking/aromatization front, zeolites, particularly ZSM‐5, were recognized early for steering oxygenates toward gasoline/jet‐range aromatics and iso‐paraffins via strong Brønsted acidity and shape selectivity.^[^ ^11^ ^]^ However, single‐crystal, microporous zeolites suffered from diffusion limitations and rapid coke growth. The field responded with hierarchical zeolites (meso‐/micro‐porous ZSM‐5, Beta, Y) and desilication/dealumination protocols that introduced secondary porosity, reducing residence times and suppressing polyaromatic coke.^[^ ^12^ ^]^ Metal‐modified zeolites (Ga, Zn, Ni) added dehydrogenation function and shifted product slates, while ex situ catalytic fast pyrolysis (CFP) configurations limited catalyst contact with solids/ash, improving catalyst life.^[^ ^13^ ^]^ Beyond zeolites, mesoporous silicas (MCM‐41, SBA‐15) and amorphous silica–aluminas offered tunable acidity and larger pores for bulky oligomers, albeit with lower intrinsic shape selectivity.^[^ ^14^ ^]^

A major advance was the emergence of bifunctional catalysts that integrate metallic hydrogenation/deoxygenation sites with acid sites for cracking/isomerization either within one particle (e.g., Pt/ZrO_2_–SO_4_^2^^−^, Ni/H‐ZSM‐5) or as physically coupled beds. These systems improved hydrogen economy (by hydrogenating coke precursors before cracking), tailored jet‐range distributions (C_8_–C_16_), and balanced ring‐saturation with controlled ring‐opening to meet smoke point and freezing‐point targets.^[^ ^15^ ^]^ In parallel, carbides (Mo_2_C, WC), phosphides (Ni_2_P, MoP), and nitrides rose as sulfur‐free HDO families with noble‐metal‐like behavior for C–O bond cleavage and superior tolerance to water; advances in synthesis (temperature‐programed carburization/phosphidation, confinement) and support choice (ZrO_2_, carbon) improved stability and dispersion.^[^ ^16^ ^]^

Despite this progress, some research gaps persist, for example, catalyst deactivation by coking, hydrothermal sintering, halide poisoning, and support transformation, which continue to limit economics. There is a need for durability‐centric design (hierarchical porosity, hydrophobic surfaces, poison traps, regenerable phases) and gentle yet effective regeneration protocols (oxidative decoking with minimal dealumination, phase reactivation for phosphides/carbides) validated over many cycles.^[^ ^17^ ^]^ Another limitation is that materials sustainability and critical‐metal dependence remain underexplored; pathways to reduce or recycle PGMs, recover Ni/Mo/W, and employ earth‐abundant alternatives are crucial for scale.^[^ ^18^ ^]^

Due to these limitations, direct utilization of bio‐oil in existing energy infrastructure, such as combustion engines or jet turbines, is impractical. To overcome these challenges and improve bio‐oil quality, it must undergo further upgrading processes to remove oxygen and enhance its fuel properties. These upgrading processes aim to produce drop‐in fuels that are compatible with current transportation and energy systems, such as gasoline, diesel, or jet fuel.^[^ ^6^ ^]^

### 1.2 Importance of Converting Bio‐Oil to Biojet Fuel

The conversion of bio‐oil to biojet fuel is significant due to the urgent need for sustainable fuel alternatives in the aviation industry.^[^ ^19^ ^]^ Jet fuel is highly dependent on petroleum‐based sources and contributes substantially to carbon emissions. Biojet fuel, derived from biomass, offers a promising solution to mitigate these emissions while ensuring energy security.^[^ ^20^ ^]^ The transition to biojet fuel is crucial to environmental sustainability, meeting stringent international regulations on aviation emissions, and fostering energy independence. Unlike ground transportation, which is rapidly transitioning to electric vehicles, the aviation sector relies heavily on liquid hydrocarbon fuels due to the high energy density required for long‐distance flights. Therefore, the development of biojet fuel from renewable sources, such as biomass, is seen as a critical step toward achieving sustainable aviation.

The aviation sector is one of the fastest‐growing sources of greenhouse gases, and addressing its environmental impact is crucial to achieving international climate goals. Biojet fuel, derived from renewable biomass, offers a promising solution to mitigate these emissions while ensuring the continuity of aviation operations.^[^ ^21^ ^]^ It reduces carbon footprint and improves energy security by diversifying the fuel supply base. Biojet fuel must meet stringent specifications to ensure safety, performance, and compatibility with existing aviation infrastructure. This necessitates advanced upgrading processes to convert raw bio‐oil into a high‐quality fuel that meets these stringent requirements. The challenges in upgrading bio‐oil include the removal of oxygenated compounds, the reduction of acidity, and the improvement of thermal and chemical stability.^[^ ^5^ ^]^ Efficient catalytic processes are essential to achieve these transformations, making catalyst development a critical area of research.

### 1.3 Objectives and Scope of the Review

This review aims to provide a comprehensive analysis of the advancements in catalyst design for upgrading biomass‐derived bio‐oil to biojet fuel. It will cover the current state of bio‐oil production and its chemical composition, the challenges associated with its conversion, and the various types of catalysts that have been developed to address these challenges. The review will also consider the mechanisms by which these catalysts operate, the optimization of catalytic processes, and the performance metrics used to evaluate their efficiency. By examining recent progress and highlighting future research directions, this review seeks to contribute to ongoing efforts to develop effective, scalable, and economically viable catalytic processes for biojet fuel production.

## 2 Overview of Biomass‐Derived Bio‐Oil

### 2.1 Sources of Biomass

Biomass, a diverse category encompassing organic materials such as agricultural residues, forestry residues, energy crops like switchgrass and miscanthus, and organic wastes, serves as the primary feedstock for producing bio‐oil through thermochemical processes.^[^ ^22^ ^]^ These processes typically involve pyrolysis, thermal decomposition without oxygen, or HTL, which uses water under high temperature and pressure. Each biomass source offers distinct advantages and challenges based on its composition, availability, and regional suitability, influencing the economics and sustainability of bio‐oil production.^[^ ^23^ ^]^

**Table** **1** presents a comparison of bio‐oil properties derived from various feedstocks, with a focus on water content. The water present in bio‐oil originates both from the initial moisture in the biomass and as a byproduct of the pyrolysis process. A high‐water content is generally considered a drawback for the application of bio‐oil as a fuel, as it lowers the energy density and can cause instability. The acceptable range for the water content in bio‐oil is typically between 25 and 26 wt%. According to the data, bio‐oil obtained from sugarcane bagasse and banana stem falls within this acceptable range, indicating a better fuel potential. In contrast, bio‐oils derived from rice husk and wheat straw exhibit a higher water content, making them less suitable for direct use without additional upgrading or treatment.^[^ ^24^ ^]^

**Table 1:** Comparison of properties between bio‐oils from different feedstocks.^[^ ^24^ ^]^

Properties	Sugarcane bagasse	Rice husk	Coconut shell	Wood sawdust	Corn stover	Wheat straw	Municipal solid waste	Banana stem
Kinematic viscosity [cSt]	21.50	4.861–16.277	36	14 (cP at 40 °C)	13 (cP at 40 °C)	23.5	2.00	–
Density [kg m^−3^]	1,150	1,138–1,170	1,090	1,060	1,020	–	1,205	1,200
pH	3.85	2.85–3.2	3.15–3.28	2.07	2.64	2.4	1.5	3.18 ± 0.02
Higher heating value (HHV) [MJ kg^−1^]	23.50	14.285–21.742	38.6	20.38	20.39	24.2	13.10	7.97
Water content [%]	11.60	26.18–41.32	–	–	–	26.7	–	20.0 ± 0.1
Elemental analysis [%]								
C	52.62	37.86	75.4	24.86	13.00	50.78	40.80	9.70
H	7.40	0.21	11.7	7.17	8.08	3.20	6.29	11.21
O	39.10	5.24 ± 0.01	10.5	67.61	78.39	44.42	52.91	50.42
N	0.75	35.32	2.4	0.35	0.53	1.37	–	28.67
S	<0.07	2.15 0.68 ± 0.06	–	–	–	–	–	–

### 2.2 Bio‐Oil Production Methods

Bio‐oil production involves the conversion of biomass into liquid form through thermochemical processes. Pyrolysis, the most common method, subject's biomass to rapid heating without oxygen, decomposes organic materials into vapors, which are then condensed into bio‐oil. Various pyrolysis variations, such as fast and slow pyrolysis, offer different bio‐oil yields and qualities, influenced by factors such as temperature, residence time, and biomass feedstock.^[^ ^25^ ^]^ Fast pyrolysis involves heating biomass at high temperatures (typically between 450 and 600 °C) with very short residence times (seconds), which maximizes bio‐oil yield, often around 60–75% by weight. This method is advantageous for bio‐oil production because of its high efficiency and ability to process large amounts of biomass. However, fast pyrolysis bio‐oil tends to have a high oxygen content, leading to a lower energy density and requiring further improvements to improve its fuel properties.^[^ ^26^ ^]^ In contrast, slow pyrolysis operates at lower temperatures (around 400 °C) and longer residence times, producing a lower bio‐oil yield but higher amounts of biochar, which can be used as a carbon‐rich soil amendment. Slow bio‐oil pyrolysis is often more viscous and contains higher levels of larger and more complex molecules, making it less suitable for direct fuel applications without further refinement.^[^ ^27^ ^]^

In contrast, HTL uses water as a solvent under elevated temperatures (typically 250 to 374 °C) and pressures (up to 25 MPa) to convert biomass into a bio‐oil product with potentially higher energy content and stability.^[^ ^28^ ^]^ HTL offers several advantages over pyrolysis, including the ability to process wet biomass without predrying, making it especially suitable for feedstocks such as algae, sewage sludge, or food waste. The bio‐oil produced from HTL generally has a higher energy content, lower oxygen content, and greater stability compared to those of pyrolysis oils, because of the different reaction mechanisms involved in the process. This results in a bio‐oil that requires less extensive upgrading to become a usable fuel.^[^ ^29^ ^]^

**Figure 1:**
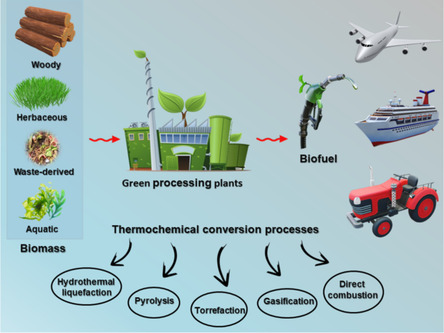
Conversion of biomass into aviation fuels through various pathways. Reproduced with permission.^[^ ^31^ ^]^ Copyright 2021, Springer Nature.

In addition to pyrolysis and HTL, direct combustion and gasification are key thermochemical conversion techniques for transforming biomass into usable energy and fuels, as shown in **Figure** **1**. Direct combustion is the simplest and most traditional method, involving burning biomass in excess oxygen to produce heat. This heat can be used directly for residential or industrial heating or to generate electricity through steam turbines. Although this method is widely applied and technologically mature, it has a relatively low energy efficiency (typically 20–30%) and releases greenhouse gases and particulate emissions unless carefully controlled.^[^ ^30^ ^]^ Additionally, combustion does not produce liquid fuels, making it unsuitable for producing biojet fuel directly, but it plays a role in integrated biorefineries as a means of utilizing residual biomass or providing process heat.

Gasification, in contrast, converts biomass into a combustible gas mixture known as syngas, mainly composed of carbon monoxide (CO), hydrogen (H_2_), carbon dioxide (CO_2_), and methane (CH4), through partial oxidation at high temperatures (typically 700–1000 °C) in a controlled environment with limited oxygen or steam. The resulting syngas can be used directly for heat and power generation or further processed by catalytic routes such as Fischer–Tropsch synthesis to produce liquid hydrocarbons, including jet fuel range alkanes.^[^ ^31^ ^]^ This pathway holds significant promise for sustainable aviation fuel (SAF) production, particularly when combined with renewable hydrogen sources. However, biomass gasification faces challenges such as feedstock variability, tar formation, and the need for extensive gas cleanup prior to catalytic upgrading. Moreover, high capital investment and technological complexity have limited the widespread commercial deployment of gasification‐based SAF routes.^[^ ^32^ ^]^

### 2.3 Chemical Composition of Bio‐Oil

Bio‐oil is a complex mixture of oxygenated hydrocarbons, phenolic compounds, aldehydes, ketones, acids, water, and various other functional groups. The composition of bio‐oil varies widely depending on the type of biomass feedstock, the thermochemical conversion method used (e.g., fast pyrolysis, HTL), and the operating conditions.^[^ ^33^ ^]^ Oxygen functional groups, such as the hydroxyl, carboxyl, and carbonyl groups, contribute to the high oxygen content and acidity of bio‐oil, which pose challenges for its direct use as a fuel.^[^ ^34^ ^]^ These chemical characteristics also influence bio‐oil stability, viscosity, and heating value, affecting its suitability for upgrading to higher‐value biofuels such as biojet fuel. The presence of phenolic compounds in bio‐oil, which are derived primarily from the lignin fraction of lignocellulosic biomass, poses a particular challenge for upgrading. Phenolics are highly reactive and tend to polymerize during storage or upgrading, leading to coke formation and catalyst deactivation.^[^ ^35^ ^]^ Aldehydes and ketones, derived from the degradation of cellulose and hemicellulose, contribute to the instability of bio‐oil, as they can undergo condensation reactions that increase viscosity and reduce the shelf life of the oil.^[^ ^36^ ^]^ Carboxylic acids, such as acetic and formic acids, are responsible for the high acidity of bio‐oil, leading to corrosion in storage tanks and processing equipment.^[^ ^37^ ^]^ Understanding the chemical composition of bio‐oil is crucial for designing effective catalytic processes that can selectively transform its components into more stable and energy‐dense fuels, thus enhancing the viability of biomass‐derived bio‐oil as a renewable energy source.^[^ ^38^ ^]^

## 3 Fundamentals of Bio‐Oil Upgrading

### 3.1 Challenges in Upgrading Bio‐Oil

Upgrading biomass‐derived bio‐oil poses several significant challenges because of its complex chemical composition and inherent properties. One of the primary challenges is the high oxygen content, typically ranging from 20% to 40% by weight, which leads to low energy density and reactivity, contributing to instability and corrosiveness. Oxygen also produces high acidity, which can catalyze undesirable reactions and degrade catalysts used in upgrading processes. Additionally, bio‐oil contains various oxygenated compounds such as aldehydes, ketones, and acids, which require selective conversion to enhance the quality and stability of the resulting biofuels. Addressing these challenges requires advanced catalytic technologies capable of selectively removing oxygen while maintaining the overall energy efficiency of the conversion process. In addition to oxygen content, thermal instability is another critical challenge in bio‐oil upgrading. Bio‐oil tends to polymerize and degrade over time, leading to increased viscosity and the formation of solids, which complicates its storage, transport, and further processing. This instability is caused by reactive compounds, such as aldehydes and unsaturated hydrocarbons, which can undergo condensation reactions. The instability of bio‐oil makes it unsuitable for direct use and requires stabilization steps before upgrading.^[^ ^39^ ^]^ Furthermore, bio‐oil often contains a significant amount of water, which can be as high as 30%, depending on the production method. This water content reduces the heating value of the bio‐oil and dilutes the hydrocarbon content, making the upgrading process less efficient. Water must be removed or managed during the upgrading process to ensure that the final fuel has a high enough energy density to meet the transportation fuel standards.^[^ ^40^ ^]^

Another issue is the presence of heteroatoms, such as sulfur and nitrogen, which are common in certain types of biomass feedstock, especially waste‐derived and algal sources. These heteroatoms can poison catalysts during upgrading processes, reducing the efficiency and life span of catalysts.^[^ ^41^ ^]^ Removal of sulfur and nitrogen from bio‐oil is critical, especially for compliance with aviation fuel standards, as the presence of these elements can lead to the formation of harmful emissions during combustion and degrade the performance of engines.^[^ ^42^ ^]^

**Figure 2:**
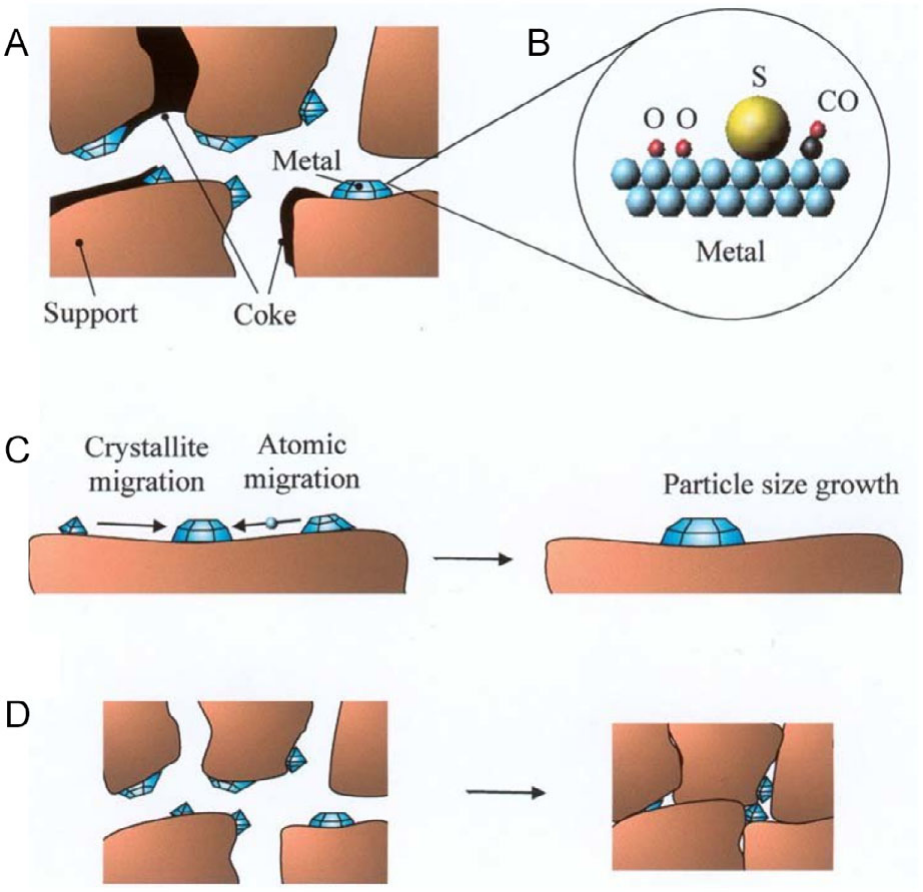
Deactivation mechanisms: A) coke formation, B) poisoning, C) sintering of the active metal particles, and D) sintering and solid–solid phase transitions of the washcoat and encapsulation of active metal particles. Reproduced with permission.^[^ ^44^ ^]^ Copyright 2003, University of Oulu.

In **Figure** **2**, the challenge of catalyst deactivation is illustrated with a three‐way deactivation mechanism. Coke formation is illustrated in Figure 2A, a common issue during bio‐oil upgrading, where carbon‐rich residues accumulate on the catalyst surface due to thermal decomposition or polymerization of heavy hydrocarbons and oxygenates. This carbon buildup can block active sites and pores, reducing catalytic activity, selectivity, and efficiency. High reaction temperatures, long residence times, and feedstocks rich in unsaturated or aromatic compounds often promote coke formation.^[^ ^43^ ^]^ Poisoning of a three‐way catalyst because of the accumulation of impurities on the active sites (see Figure 2B) is typically a slow and irreversible phenomenon. The accumulation of poisons on the active sites blocks the access of reactants to these active sites. As a result of poisoning, catalytic activity may decrease without affecting selectivity, but selectivity can also often change, as some of the active sites are deactivated while others are practically unaffected.^[^ ^44^ ^]^ Sintering, as shown in Figure 2C,D, refers to the reduction of the active surface area of a catalyst caused by the growth of crystals, either from the bulk material or the active phase. In supported metal catalysts, this loss of active surface is typically due to the agglomeration and coalescence of small metal particles into larger crystallites. Sintering can occur through two primary mechanisms. The first, known as the atomic migration model, involves metal atoms moving from one crystallite to another via the surface or gas phase, leading to the shrinking of smaller particles and the growth of larger ones. The second mechanism involves the movement of entire crystallites across the surface, where they collide and merge, further contributing to the formation of larger particles and consequently the decline in catalytic activity.^[^ ^44^ ^]^

Finally, to produce aviation fuel, bio‐oil must undergo significant refining to meet strict standards for jet fuel. Aviation fuel requires a high energy density, specific chemical properties (such as a suitable boiling range and low freezing point), and strict safety and performance criteria.^[^ ^45^ ^]^

**Figure 3:**
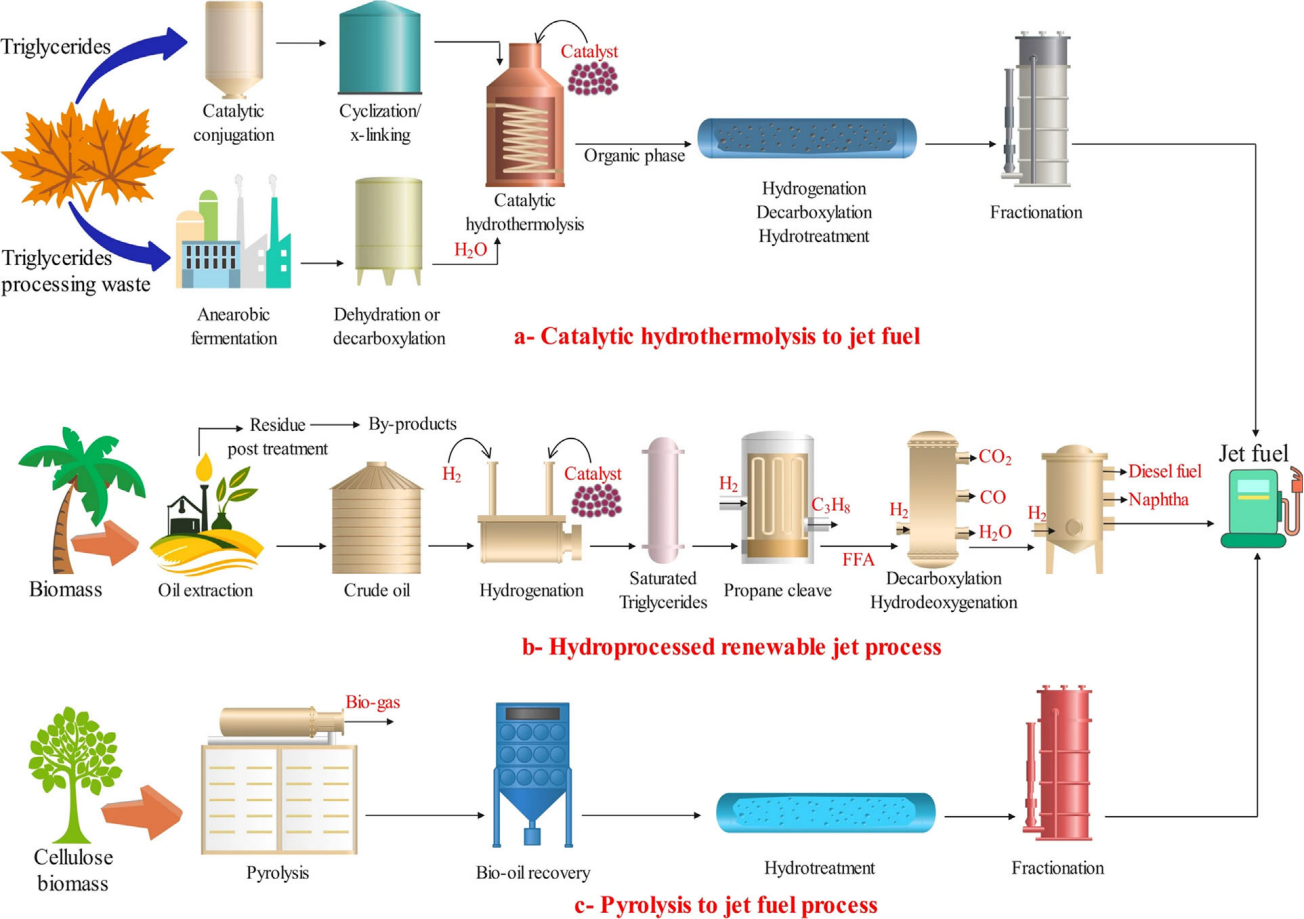
Key pathways for converting oil into biojet fuel. Reproduced with permission.^[^ ^120^ ^]^ Copyright 2025, Springer Nature.

**Figure** **3** shows three key pathways for the conversion of oil to jet fuel. In catalytic hydro thermolysis (a), oil triglycerides are converted to jet fuel using a solvent‐based method that involves a combination of catalyst, heat, and water under high pressure. The high temperature and pressure promote the breakdown of oil molecules into smaller hydrocarbon fragments. These fragments are then rearranged and converted into hydrocarbons with the aid of a catalyst. The resulting mixture is further purified to meet the specifications required for jet fuel.

The hydroprocessed renewable jet (b) process involves several stages. Initially, the feedstock oil is subjected to hydroprocessing, where it is mixed with hydrogen and passed through a catalyst bed inside a hydrotreating reactor operating at elevated temperature and pressure. This process removes impurities such as sulfur, nitrogen, and oxygen while saturating unsaturated hydrocarbons by hydrogenation. The hydrotreated oil is then subjected to hydrocracking, a catalytic process that breaks down larger hydrocarbon molecules into smaller and more valuable ones suitable for jet fuel.

The pyrolysis to jet fuel (c) strategy begins with the pyrolysis of oil feedstock, a thermal degradation process carried out in the absence of oxygen. This breaks down large hydrocarbon molecules into smaller and more volatile compounds. The products of pyrolysis include bio‐oil, gases, and char. The bio‐oil portion is further upgraded through processes such as hydrotreatment and hydrocracking to remove impurities, enhance stability, and adjust its chemical composition to meet jet fuel standards. Finally, the upgraded bio‐oil is refined and fractionated to isolate the components suitable for jet fuel production.

The abbreviations used include H_2_O (water), H_2_ (hydrogen), C_3_H_8_ (propane), free fatty acids (FFA), CO (carbon monoxide), and CO_2_ (carbon dioxide).

### 3.2 Desired Properties of Biojet Fuel

Biojet fuel must meet stringent specifications to ensure compatibility with existing aviation infrastructure, engines, and safety standards. Key properties include high energy density, low freezing point, low viscosity, and compatibility with existing jet fuel formulations. Biojet fuel should also exhibit high thermal stability and resistance to degradation during storage and transport, ensuring reliability and performance in aviation operations. Achieving these properties through effective bio‐oil upgrading processes is critical for the widespread adoption of biojet fuel as a sustainable alternative to conventional jet fuels derived from fossil sources.^[^ ^46^ ^]^ Aviation fuel is a clear, straw‐colored liquid that is primarily used in compression ignition engines. Conventional aviation turbine fuel, known as kerosene, is composed of a complex mixture of hundreds of hydrocarbons, typically derived from the middle distillate fraction of crude oil. It represents approximately 10% of the total crude oil production, with the remaining majority allocated to gasoline and diesel production. Regarding the range of hydrocarbons, jet fuel falls between gasoline and diesel: gasoline is lighter and more volatile, while diesel is heavier and more susceptible to waxing at low temperatures.^[^ ^46^ ^]^ **Table** **2** provides key comparisons of properties among these three fossil‐based liquid fuels. Jet A‐1 is the standard fuel used in commercial aviation, characterized by a carbon number range of C_8_ to C_16_. This range is carefully controlled during the refining process to ensure that the fuel meets specific performance and safety standards.

**Table 2:** The general comparison of petroleum gasoline, aviation fuel, and diesel fuel properties.^[^ ^46^ ^]^

Fuel properties	Gasoline	Aviation fuel	Diesel
Density at 15 °C [g cm^−3^]	0.72–0.78	0.75–0.84	0.82–0.85
Kinematic viscosity [mm^2^ s^−1^]	0.37–0.44 (at 20 °C)	Max. 8 (at −20 °C)	2.00–4.50 (at 40 °C)
Lower heating value [MJ kg^−1^]	43.4	43.0	43.4
Flashpoint [°C]	−43	Min. 38	Min. 55
Boiling point [°C]	Max. 210 (100% recov.)	Max. 300 (100% recov.)	Max. 360 (100% recov.)
Cloud point [°C]	−57	–	−15 to 5
Pour point [°C]	–	–	−35 to −15
Freezing point [°C]	–	Min. −47 (Jet A‐1)	–

### 3.3 General Upgrading Pathways (e.g., Hydroprocessing, Catalytic Cracking)

Several pathways are employed to upgrade bio‐oil to enhance its quality and suitability for use as a biojet fuel. Hydrodeoxygenation (HDO) is a key catalytic process for upgrading bio‐oil by removing oxygen in the form of water, making the bio‐oil more suitable for fuel applications. The mechanism of HDO involves the use of hydrogen to break down functional groups containing oxygen, such as the hydroxyl, carbonyl, and carboxyl groups, converting them into hydrocarbons.^[^ ^15^ ^]^ Hydrogen plays a critical role in this process, as it not only helps remove oxygen but also stabilizes the resulting hydrocarbons, preventing further polymerization and improving the fuel quality. This pathway aims to increase the content of hydrocarbons and reduce oxygen levels, thus improving the energy density and stability of the resulting biofuels.^[^ ^47^ ^]^ The catalysts typically used in HDO processes are sulfided metal catalysts, such as NiMo (nickel‐molybdenum) and CoMo (cobalt‐molybdenum), which are supported on materials like alumina to provide high surface area and stability under reaction conditions.^[^ ^48^ ^]^

Catalytic cracking, another pathway, involves breaking down larger molecules in bio‐oil into smaller, more valuable hydrocarbons using acid catalysts. This process helps to adjust the molecular weight distribution and reduce the oxygen content, improving the overall quality of biojet fuel.^[^ ^49^ ^]^ Catalytic cracking uses specialized catalysts, such as zeolites or mesoporous materials, to guide the reaction toward producing desired hydrocarbons while minimizing byproducts. Zeolites, with their well‐defined pore structures and strong acidity, are particularly effective for this process, promoting cleavage of C–C bonds and facilitating the formation of gasoline‐range hydrocarbons.^[^ ^50^ ^]^

**Figure 4:**
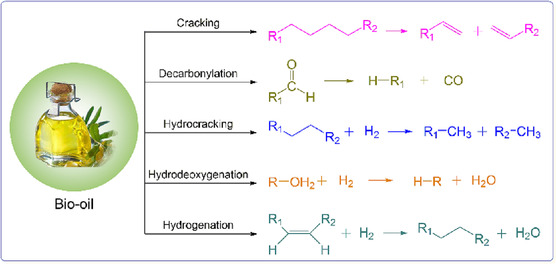
Some of the different methods of bio‐oil upgrading and modification. Reproduced with permission.^[^ ^121^ ^]^ Copyright 2023, Elsevier B.V.

Decarboxylation and decarbonylation are catalytic processes that remove oxygen from bio‐oil molecules without hydrogen. These processes remove oxygen in the form of carbon dioxide (decarboxylation) or carbon monoxide (decarbonylation) rather than water, as in HDO. Some of these pathways are shown in **Figure** **4**. These methods are attractive because they eliminate the need for expensive hydrogen, which can be a limiting factor in the HDO process.^[^ ^51^ ^]^ Typical catalysts for these processes include noble metals such as palladium and transition metals such as nickel or ruthenium, supported on materials such as carbon or silica. While these processes effectively reduce the oxygen content of bio‐oil, they typically result in some loss of carbon atoms, which can reduce the overall fuel yield. This trade‐off between oxygen removal and carbon loss makes decarboxylation and decarbonylation less efficient for producing high hydrocarbon fuel yields than HDO.^[^ ^52^ ^]^ Each upgrading pathway offers distinct advantages and challenges, requiring customized catalysts and process conditions to optimize bio‐oil conversion efficiency and product quality for SAF applications.

## 4 Catalyst Design for Bio‐Oil Upgrading

### 4.1 Catalytic Mechanisms and Principles

Catalytic upgrading of biomass‐derived bio‐oil involves complex chemical transformations to remove oxygen‐containing functional groups and adjust molecular weight distributions to improve fuel properties. The catalytic mechanisms typically include reactions such as hydrogenation, hydrodeoxygenation (HDO), decarboxylation, and decarbonylation, which are facilitated by active sites on catalyst surfaces. Hydrogenation and HDO, for instance, involve the addition of hydrogen to oxygenated compounds, leading to their conversion into water and hydrocarbons, respectively. The decarboxylation and decarbonylation reactions remove carboxyl and carbonyl groups to reduce acidity and enhance stability. Understanding these mechanisms is crucial to designing catalysts that can selectively promote desired reactions while minimizing side reactions and catalyst deactivation.^[^ ^15^ ^]^ Bio‐oil typically contains oxygenated compounds such as alcohols, aldehydes, ketones, carboxylic acids, esters, and phenolics, which must be converted into hydrocarbons to meet jet fuel standards. Two critical processes in this transformation are oxidation (removing oxygen‐containing functionalities through controlled reactions) and deoxygenation (eliminating oxygen in the form of H_2_O, CO, or CO_2_).^[^ ^53^ ^]^

The oxidation mechanism in upgrading is generally aimed at stabilizing bio‐oil by converting reactive species (such as aldehydes and phenols) into less reactive intermediates. Partial oxidation can improve storage stability by reducing polymerization and coke formation. However, excessive oxidation decreases the energy content of the fuel. Thus, oxidation is often considered a pretreatment or conditioning step rather than the main upgrading pathway.^[^ ^54^ ^]^

The deoxygenation mechanism, in contrast, is central to hydrodeoxygenation (HDO), catalytic cracking, and decarboxylation/decarbonylation processes. These mechanisms proceed through several distinct pathways:

In hydrodeoxygenation (HDO), oxygen is removed as water via hydrogen addition, a reaction that typically requires bifunctional catalysts such as NiMo/Al_2_O_3_, CoMo/Al_2_O_3_, or noble metals including Pd, Pt, and Ru. The mechanism involves the hydrogenation of C=O bonds to alcohol intermediates, followed by C—O bond cleavage, ultimately producing hydrocarbons and H_2_O. This pathway is especially effective for generating aviation‐range alkanes with high hydrogen content and excellent fuel properties, though it demands substantial hydrogen input.^[^ ^55^ ^]^ In contrast, decarboxylation (DCO_2_) removes oxygen as CO_2_ and generally operates over catalysts such as Pd/C, Pt/C, or mixed oxides. While this mechanism has the advantage of avoiding the high hydrogen consumption required in HDO, it sacrifices carbon yield, which can be a limitation when maximizing hydrocarbon production.^[^ ^56^ ^]^ Decarbonylation (DCO) removes oxygen in the form of CO and often occurs in parallel with DCO_2_ using similar catalytic systems. This pathway is selective for oxygenates such as aldehydes and carboxylic acids, but excessive CO formation can pose challenges for process integration, as it necessitates effective gas management strategies. Together, these mechanisms highlight the trade‐offs between hydrogen demand, carbon efficiency, and process complexity in selecting the most appropriate catalytic route for bio‐oil upgrading.^[^ ^57^ ^]^

The choice of catalyst is directly tied to the dominant mechanism desired. For example, sulfided NiMo or CoMo catalysts are widely used in industrial HDO because of their high activity and robustness, but they require continuous sulfur feed to maintain activity.^[^ ^58^ ^]^ Noble metals (Pd, Pt, Ru), though more expensive, exhibit superior resistance to deactivation and allow operation under milder conditions.^[^ ^59^ ^]^ Meanwhile, zeolite‐based catalysts (ZSM‐5, Beta, *Y*) promote cracking and aromatization, producing hydrocarbons in the aviation fuel range but often face challenges with coke deposition due to strong acidity.^[^ ^60^ ^]^ Emerging materials such as metal–organic frameworks (MOFs), mixed metal oxides (MMOs; CeO_2_‐ZrO_2_, TiO_2_‐Al_2_O_3_), and phosphide/nitride catalysts are also gaining attention for their ability to tailor reaction pathways and improve selectivity while mitigating deactivation.^[^ ^61^ ^]^ Ultimately, a deeper mechanistic understanding is crucial not only for selecting the appropriate catalyst but also for designing multifunctional systems that balance activity, selectivity, hydrogen efficiency, and stability. For instance, bifunctional catalysts that integrate metallic sites (for hydrogenation/deoxygenation) with acidic sites (for cracking/isomerization) are proving effective in achieving high‐quality biojet fuels.^[^ ^62^ ^]^

### 4.2 Criteria for Effective Catalysts in Bio‐Oil Upgrading

Effective catalysts for bio‐oil upgrading must meet several criteria to ensure optimal performance and economic viability. Key considerations include high catalytic activity and selectivity towards target reactions, stability under harsh operating conditions, high temperatures and pressures, and resistance to catalyst poisoning or deactivation by bio‐oil components like sulfur and nitrogen compounds.^[^ ^63^ ^]^ Catalysts should also allow efficient conversion of a wide range of bio‐oil constituents while minimizing energy consumption and environmental impact.^[^ ^64^ ^]^ Moreover, catalysts that facilitate easy separation and recycling and reduce overall process costs and waste generation are preferred for commercial scale biojet fuel production. In addition, catalysts with high surface area and well‐dispersed active metal sites facilitate efficient reaction kinetics, as these features improve accessibility and reduce the likelihood of deactivation.^[^ ^65^ ^]^ These combined properties are essential to achieve the stringent standards required to produce biojet fuel. When these criteria are met, catalysts play a pivotal role in enabling efficient and sustainable conversion of biomass‐derived bio‐oil into high‐quality biojet fuels, advancing the viability of renewable energy sources in aviation and beyond.

**Table** **3** is deduced from the literature reviewed by Sun et al. *2025.* The table categorizes different types of catalysts, zeolites, metal oxides, metal‐supported catalysts, and phosphates, highlighting their respective advantages and drawbacks. Zeolites are known for their high acidity, which makes them effective in acid‐catalyzed reactions such as cracking and isomerization. However, they suffer from pore size limitations, which can restrict access for larger bio‐oil molecules and reduce their overall effectiveness.^[^ ^66^ ^]^ Metal oxides offer excellent thermal stability, making them well‐suited for high‐temperature applications. However, their main limitation is their generally lower acidity, which can hinder their performance in reactions requiring strong acid sites. Metal‐supported catalysts are highly active because of the presence of dispersed metal particles that facilitate hydrogenation and deoxygenation. Despite their activity, these catalysts are prone to metal sintering at elevated temperatures, which decreases their efficiency over time. Carbonaceous materials, such as activated carbon and biochar, have emerged as promising catalyst supports or active materials because of their high surface area, tunable surface chemistry, and sustainability. They can be derived from biomass and customized for specific catalytic functions, offering a renewable and cost‐effective alternative. Their main drawbacks include lower intrinsic catalytic activity compared to metal‐based catalysts and potential stability issues under harsh reaction conditions. Together, these catalyst types offer a variety of properties and trade‐offs that must be carefully considered when designing an efficient pathway to convert bio‐oil into jet fuel.^[^ ^66^ ^]^

**Table 3:** The advantages and drawbacks of different types of catalysts.

Catalyst type	Advantages	Drawbacks
Zeolites	Highly selective, shape‐selective, high‐thermal stability, good mechanical strength, versatile applications (adsorption, catalysis, separation)	Can have diffusion limitations due to microporous nature, potential for deactivation, and synthesis can be complex
Metal oxides	Versatile, wide range of applications (oxidation, reduction, environmental remediation), tunable properties through doping and composition	May require high temperatures for activity, potential for sintering, can be environmentally problematic (e.g., heavy metal oxides)
Metal‐supported catalysts	High catalytic activity, can be easily modified to enhance selectivity, and excellent control over reaction pathways	Often require specific reaction conditions, can be expensive, and have the potential for leaching into the reaction mixture
Carbonaceous materials	Abundant, inexpensive, can be modified to enhance selectivity and stability	Can have low surface area, may not be as selective as other materials, may require pretreatment

### 4.3 Types of Catalysts Used

#### 4.3.1 Heterogeneous Catalysts

Heterogeneous catalysts are widely used in bio‐oil upgrading because they facilitate reactions at the interface between solid catalyst surfaces and liquid bio‐oil phases.^[^ ^67^ ^]^ Common heterogeneous catalysts include metal‐based catalysts such as nickel (Ni), palladium (Pd), and platinum (Pt), which are effective in hydrogenation and HDO reactions. In addition, solid acid catalysts, such as zeolites and MMOs, are used for acid‐catalyzed reactions such as esterification and dehydration.^[^ ^68^ ^]^ The choice of a heterogeneous catalyst depends on factors such as reaction specificity, stability under operating conditions, and ease of catalyst separation and recycling, which are critical for commercial viability.

#### 4.3.2 Homogeneous Catalysts

Homogeneous catalysts, on the contrary, are fully dissolved in the bio‐oil feedstock, facilitating uniform catalytic activity throughout the reaction mixture.^[^ ^69^ ^]^ Examples include transition metal complexes and organometallic compounds, which offer precise control over the selectivity and efficiency of the reaction because of their defined molecular structures and tunable properties. Homogeneous catalysts are particularly advantageous for complex transformation reactions and can exhibit catalytic activity higher than that of their heterogeneous counterparts. However, challenges such as catalyst separation and recycling and potential contamination of the product with catalyst residues must be addressed for their practical application in large‐scale biojet fuel production.^[^ ^70^ ^]^

## 5 Heterogeneous Catalysts

### 5.1 Metal‐Based Catalysts

#### 5.1.1 Noble Metals (e.g., Pt, Pd)

Noble metal catalysts, such as platinum (Pt) and palladium (Pd), are highly effective in catalyzing hydrogenation and hydrodeoxygenation (HDO) reactions in bio‐oil upgrading processes.^[^ ^71^ ^]^ These metals possess unique catalytic properties because of their ability to adsorb hydrogen atoms and facilitate the breaking of carbon‐oxygen bonds in oxygenated compounds present in bio‐oil. Pt and Pd catalysts are known for their high activity and selectivity, particularly in converting phenolic compounds and aldehydes/ketones into corresponding alkanes and alcohols. However, their high cost and susceptibility to deactivation by sulfur and nitrogen compounds in bio‐oil require careful catalyst design and operational conditions to maintain long‐term stability and efficiency.^[^ ^72^ ^]^

#### 5.1.2 Transition Metals (e.g., Ni, Co, Mo)

Transition metal catalysts, including nickel (Ni), cobalt (Co), and molybdenum (Mo), are widely used in the upgrade of bio‐oil due to their robust catalytic activity and relative affordability compared to noble metals.^[^ ^73^ ^]^ Ni‐based catalysts, for instance, are effective in hydrogenation and cracking reactions, converting oxygenated species into hydrocarbons suitable for biojet fuel production. Co and Mo catalysts are known for their versatility in catalyzing various reactions, such as hydrodeoxygenation and decarboxylation, which are essential for reducing acidity and improving bio‐oil stability. These transition metal catalysts can be customized through formulation and support materials to enhance selectivity and durability under harsh operating conditions, making them promising candidates for industrial‐scale bio‐oil upgrading processes.^[^ ^74^ ^]^

### 5.2 Zeolite‐Based Catalysts

#### 5.2.1 Types of Zeolites

Zeolite‐based catalysts are renowned for their acidic properties and well‐defined pore structures, enabling them to catalyze acid‐catalyzed reactions in bio‐oil upgrading selectively.^[^ ^75^ ^]^ Zeolites are particularly useful for breaking down larger molecules into hydrocarbons within the desired fuel range. Their structural properties, such as pore size, framework type, and acidity, allow for precise control of reaction pathways.^[^ ^76^ ^]^ The acidity of zeolites is especially important in catalytic cracking, as it facilitates the breakdown of complex oxygenated compounds in bio‐oil.^[^ ^60^ ^]^ Common types include ZSM‐5, Beta zeolite, and HZSM‐5, each offering specific advantages based on pore size distribution, acidity strength, and thermal stability.ZSM‐5 zeolite, for example, is effective in catalyzing dehydration and oligomerization reactions, while Beta zeolite exhibits superior activity in cracking and isomerization processes. The choice of zeolite type depends on the desired reaction pathway and the composition of the bio‐oil feedstock, highlighting the importance of catalyst characterization and optimization for achieving desired fuel properties. Recent advances have focused on modifying zeolites to improve their stability and reduce coking, such as by adjusting pore sizes or adding secondary metal components to increase selectivity and prolong the catalyst life. Modified zeolites, including hierarchical zeolites with enhanced porosity, offer improved mass transfer, which is essential for the efficient processing of heavy molecules found in bio‐oil.^[^ ^77^ ^]^

#### 5.2.2 Zeolite Modifications for Improved Performance

To enhance catalytic performance, zeolites undergo modifications such as ion exchange, framework doping, and surface functionalization.^[^ ^78^ ^]^ These modifications aim to adjust acidity levels, improve thermal stability and resistance to coke formation, and enhance catalytic selectivity toward target reactions in bio‐oil upgrading.^[^ ^79^ ^]^ For example, ion exchange replaces exchangeable cations with the desired metal ions to tailor the acidity and catalytic activity. Framework doping introduces heteroatoms such as aluminum or silicon to alter the pore size and acidity distribution, optimizing catalytic sites for specific bio‐oil components. Surface functionalization with organic or inorganic species further enhances stability and resistance to deactivation, prolonging catalyst life under harsh operating conditions. Introducing metal dopants such as nickel, cobalt, or noble metals (e.g. platinum) into zeolite frameworks has improved catalytic activity and selectivity by promoting deoxygenation and cracking reactions.^[^ ^80^ ^]^ Tailoring the acidity of zeolites, either by altering the Si/Al ratio or through chemical treatments, has allowed better control over reaction pathways, reducing undesired by‐products. Novel zeolite frameworks, such as ZSM‐5 variants with modified pore structures, have shown promise in the handling of complex and diverse oxygenated compounds in bio‐oil.^[^ ^81^ ^]^ Collectively, these advances aim to enhance the efficiency and durability of zeolites in upgrading bio‐oil into high‐quality hydrocarbon fuels. These advances in zeolite modification contribute to their growing importance in sustainable biojet fuel production, offering customized solutions for efficient biomass conversion.

### 5.3 MMOs and Composites

MMOs and composite catalysts combine the advantages of multiple metal species and support materials to synergistically improve catalytic performance in bio‐oil upgrading.^[^ ^82^ ^]^ MMOs, such as NiMoO_4_, CoMoO_4_, and CeZrO_2_, combine the functionalities of individual oxides, increasing the activity in the removal of oxygen and hydrocarbon formation. These materials exhibit strong redox properties, tunable acid–base characteristics, and high thermal stability, making them effective for hydrodeoxygenation (HDO), catalytic cracking, and reforming. Composites incorporating both acidic and metallic functionalities further enable dual functionality catalysis, facilitating sequential or simultaneous reactions required for comprehensive bio‐oil upgrading.^[^ ^83^ ^]^ Recent advances focus on optimizing metal loading, dispersion, and interaction with support materials to maximize catalytic efficiency and durability, thereby advancing the feasibility of bio‐oil conversion technologies at commercial scales. However, these catalysts come with notable limitations. Thermal stability and structural integrity can be compromised under the high temperatures and pressures required for bio‐oil upgrading, leading to degradation over time. Additionally, coking and poisoning from impurities in bio‐oil, such as sulfur and nitrogen, can significantly reduce catalytic activity and lifespan.^[^ ^83^ ^]^ The cost of some MMOs, especially those containing rare earth elements such as cerium, is another constraint for large‐scale applications. Furthermore, it remains challenging to ensure a uniform dispersion of active components in composites, as poor dispersion can lead to uneven activity and localized deactivation.^[^ ^84^ ^]^

## 6 Homogeneous Catalysts

### 6.1 Acidic and Basic Catalysts

Acidic catalysts, such as sulfuric acid, phosphoric acid, and Lewis acids, are commonly used in reactions such as esterification, dehydration, and cracking of oxygenated compounds in bio‐oil.^[^ ^85^ ^]^ These catalysts are effective in breaking down large molecules and facilitating the removal of oxygen. On the contrary, basic catalysts, including sodium hydroxide and potassium carbonate, are particularly useful for transesterification and decarboxylation processes. Basic catalysts can neutralize acidic components in bio‐oil, improving stability and reducing corrosion risks.^[^ ^86^ ^]^ Although both types of catalyst are effective, their corrosive nature and the difficulty in separating them from the reaction products are significant limitations.

### 6.2 Organometallic Catalysts

Organometallic catalysts, which contain metal centers bonded to organic ligands, offer exceptional selectivity for targeted reactions in bio‐oil upgrading, such as hydrodeoxygenation (HDO) and hydrogenation.^[^ ^87^ ^]^ Catalysts such as ruthenium or palladium complexes are highly efficient in promoting bond‐breaking and oxygen‐removal reactions. Their tunability through ligand modification allows researchers to optimize reaction conditions and product distribution. However, organometallic catalysts are often expensive, sensitive to impurities in bio‐oil, and can degrade under harsh conditions, limiting their scalability for industrial applications.^[^ ^88^ ^]^

### 6.3 Solvent Effects on Catalytic Performance

The choice of solvent significantly affects the efficiency and selectivity of homogeneous catalytic reactions.^[^ ^89^ ^]^ Polar solvents, such as water or alcohol, can enhance the solubility of bio‐oil components and facilitate reactions like hydrolysis and hydrogenation. Nonpolar solvents, on the other hand, may stabilize intermediates and improve product separation.^[^ ^90^ ^]^ Additionally, ionic liquids have emerged as promising solvents for bio‐oil upgrading, offering tunable properties and compatibility with homogeneous catalysts. However, the high cost and environmental impact of some solvents and the challenges in recovery and recycling remain key concerns.^[^ ^91^ ^]^

## 7 Comparative Analysis of Catalysts

A comprehensive comparison of catalysts used in bio‐oil upgrading highlights their strengths, limitations, and suitability for different processes, providing valuable insights for optimizing biojet fuel production.

### 7.1 Performance Comparison of Different Catalysts

Catalyst performance is typically evaluated based on activity, selectivity, stability, and resistance to deactivation. Metal‐based catalysts, such as NiMo and Pt, exhibit excellent activity in hydrodeoxygenation (HDO) and hydrogenation reactions, effectively removing oxygen and producing high‐quality hydrocarbons.^[^ ^62^ ^]^ However, while highly effective, noble metals are expensive and prone to deactivation by sulfur and nitrogen impurities.^[^ ^92^ ^]^ With their strong acidity and structured porosity, zeolite‐based catalysts excel in catalytic cracking and selective hydrocarbon production, but are often susceptible to coking.^[^ ^93^ ^]^ On the contrary, carbon‐based catalysts and MMOs provide cost‐effective alternatives with good thermal stability and tunability. However, they may lack the same level of catalytic efficiency as metal‐supported systems.^[^ ^94^ ^]^ Homogeneous catalysts, such as organometallic complexes and acidic systems, deliver unmatched selectivity but face challenges in recovery and reuse. **Table** **4** compares nanostructured catalysts, acidity / basicity‐tuned catalysts, and hybrid catalysts in terms of their efficiency, stability, and reusability. Nanostructured catalysts are characterized by their high surface area, which enhances catalytic performance, though they can be prone to aggregation. Catalysts with tuned acidity or basicity offer improved selectivity, but achieving optimal performance requires precise control over doping levels. Hybrid catalysts combine multiple functional properties, delivering both enhanced stability and dual catalytic activity; however, their synthesis tends to be more complex and costly.

**Table 4:** Performance comparison of different catalyst optimization strategies.^[^ ^122^ ^]^

Catalyst type	Key advantages	Key disadvantages	Performance trends
Nanostructured catalysts (e.g., TiO_2_, ZnO)	High surface areaEnhanced reactant diffusionMinimal mass transfer limitations Increased active site exposure	Prone to aggregation over time High production cost	Achieves 96–98% biodiesel yield over 5–7 cycles Retains high thermal stability but may require doping for improved longevity
Acidity/basicity‐tuned catalysts (e.g., MgO, sulfonated zeolites)	Selective for specific reactions (acidic for esterification, basic for transesterification) Improves catalyst selectivity and conversion efficiency	Requires precise control of doping to balance acid/base properties Can undergo leaching of active sites in harsh conditions	Sulfonated zeolites retain 90–94% catalytic efficiency over 4–6 cycles MgO–Zeolite hybrids maintain 93–96% yield over 7–10 cycles
Hybrid catalysts (e.g., TiO_2_–zeolite, MgO‐LDH)	Dual functionality (acid–base synergy) Enhanced stability and prolonged reusability Better resistance to coke formation	More complex synthesis Higher cost compared to single‐component catalysts	TiO_2_/SiO_2_ composites maintain 95–97% yield over 5–8 cycles Hybrid MgO‐Zeolite composites retain high stability even after 10 cycles

As part of recent advancements in catalyst development for bio‐oil upgrading, various novel catalytic systems have been investigated, each offering distinct advantages in selectivity, conversion efficiency, and stability. **Table** **5** summarizes the performance of several promising catalyst types. Ni‐based catalysts supported on zeolites exhibit high selectivity for aromatic compounds, owing to the acidic and porous nature of the zeolite framework. However, coke formation often compromises their stability, which gradually deactivates the catalyst. The Mo or Co catalysts supported on alumina show high conversion efficiency and good hydrocarbon selectivity and are widely used due to their thermal stability and regenerative capability under industrial conditions.^[^ ^95^ ^]^ Bifunctional catalysts such as Pt‐ZrO_2_ combine strong metal and acidic sites, leading to excellent performance in both deoxygenation and cracking reactions, with very high conversion rates and robust stability.^[^ ^59^ ^]^ MOFs, due to their tunable porosity and active sites, offer promising selectivity, although their conversion efficiency and moisture sensitivity remain concerns, limiting their long‐term applicability.^[^ ^96^ ^]^

**Table 5:** Comparison of catalysts used for bio‐oil upgrading based on key performance indicators.

Catalyst type	Selectivity	Conversion rates	Stability	References
Ni‐based on zeolite support	High for aromatics	Up to 87%	Moderate (deactivates via coking)	[123]
Mo/Co on alumina	Good for hydrocarbons	Up to 75%	High (with periodic regeneration)	[124]
Bifunctional Pt‐ZrO_2_	Excellent (oxygen removal + cracking)	Up to 84%	High	[125]
MOF	Tunable, good selectivity	Up to 73%	Low to Moderate (sensitive to moisture)	[126]
Biochar‐supported metal	Moderate	Up to 65%	High (stable under mild conditions)	[127]
Phosphate mesoporous silica	High for deoxygenation	Up to 78%	Good (water‐tolerant)	[128]

Humidity sensitivity is a critical limitation in the application of MOFs as catalysts for bio‐oil upgrading, primarily because bio‐oil contains high water content (15–30 wt%) and generates additional water during upgrading reactions such as hydrodeoxygenation (HDO). Many MOFs, particularly those with carboxylate linkers like MOF‐5 or HKUST‐1, are prone to structural degradation when exposed to moisture.^[^ ^97^ ^]^ Water molecules can hydrolyze metal–ligand bonds, leading to framework collapse, reduced crystallinity, and significant loss of surface area. In addition, water can flood the pores, block reactant access to active sites, or strongly adsorb on Lewis acid or base sites, thereby outcompeting oxygenates and diminishing catalytic performance.^[^ ^98^ ^]^ To address these challenges, research has focused on developing water‐stable MOFs and modification strategies. For instance, Zr‐based frameworks such as UiO‐66 and MIL‐101(Cr) show superior hydrolytic stability due to robust metal–oxygen bonds, while hydrophobic functionalization of linkers with groups like –CF_3_ or –CH_3_ enhances water resistance by repelling moisture from pore surfaces.^[^ ^99^ ^]^ Other promising approaches include post‐synthetic modifications (e.g., polymer or silica coatings), embedding MOFs into hydrophobic composites such as carbon‐based matrices, and defect engineering to reduce water‐sensitive sites. Notably, ZIF‐8, with imidazolate linkers resembling zeolite structures, exhibits higher hydrolytic stability than conventional carboxylate MOFs, while UiO‐66 derivatives have been successfully applied in aqueous‐phase upgrading of oxygenates. These strategies highlight the potential of engineering humidity‐resistant MOFs for sustainable catalytic upgrading of bio‐oil; however, their scalability and economic feasibility must be carefully assessed to ensure that enhanced stability justifies the additional synthesis or modification costs in industrial biojet fuel production.^[^ ^100^ ^]^

Biochar‐supported metal catalysts present a more sustainable option with moderate conversion and selectivity. Their high thermal and chemical stability under milder reaction conditions makes them attractive for low‐cost, scalable processes.^[^ ^101^ ^]^ Lastly, phosphated mesoporous silica catalysts provide high deoxygenation selectivity and are particularly valued for their water tolerance, addressing a common challenge in processing bio‐oils with high moisture content. These findings underscore the importance of balancing activity, selectivity, and durability in catalyst design, while also considering economic and environmental factors for future biorefinery applications.

### 7.2 Trade‐Offs and Practical Considerations

Selecting a catalyst for bio‐oil upgrading involves balancing trade‐offs between performance, cost, and process requirements. Noble metal catalysts deliver superior results, but they are costly and are often reserved for specialized applications. As a result of deactivation, base metals like nickel and cobalt are more economical, but may require more frequent regeneration. Zeolite catalysts are attractive for cracking processes, yet their tendency to coke formation requires periodic regeneration, increasing operational complexity.^[^ ^102^ ^]^ The use of heterogeneous catalysts simplifies separation and recycling in comparison to homogeneous systems, but the latter offers higher precision for complex reactions. Additionally, environmental and sustainability considerations, such as the use of nontoxic and recyclable materials, increasingly influence catalyst development and selection.^[^ ^103^ ^]^

### 7.3 Case Studies of Successful Catalyst Applications

Recent case studies demonstrate how advances in catalyst design have effectively enhanced bio‐oil upgrading processes, showcasing promising industrial and research outcomes.

For example, hydrodeoxygenation (HDO) using NiMo/*γ*‐Al_2_O_3_ catalysts has achieved high efficiency in oxygen removal, producing hydrocarbon fractions compatible with aviation fuel. Modifications to these catalysts, such as incorporation of promoters such as phosphorus or cobalt, have further improved stability and activity in continuous operations.^[^ ^104^ ^]^

Modified zeolites, particularly hierarchical ZSM‐5, have been used for the catalytic cracking of bio‐oil. These zeolites demonstrated reduced coke formation and higher yields of valuable light hydrocarbons, a crucial step toward the production of fuels and chemicals from renewable sources. Advanced designs such as nanoscale zeolites with custom porosities have been particularly effective in optimizing mass transfer and reaction kinetics.^[^ ^50^ ^]^ Bifunctional metal/zeolite catalysts, such as Co‐Zn/HZSM‐5, have shown exceptional performance by harnessing the synergistic effects of metal and acid sites, improving deoxygenation and cracking reactions.^[^ ^105^ ^]^ Studies have reported a higher bio‐oil quality and yield when using these advanced configurations. In addition, palladium‐supported catalysts, combined with zerovalent metals such as zinc, have shown promise in situ hydrogen generation, stabilizing bio‐oil components, and improving overall product quality.^[^ ^55^ ^]^

In HTL, mixed metal oxide catalysts, such as CeO_2_‐ZrO_2_, have allowed effective deoxygenation under mild conditions, demonstrating both catalytic stability and reduced operational costs.^[^ ^106^ ^]^ Such catalysts, including algal feedstocks, are especially promising for wet biomass and have shown potential for scaling up. Novel approaches employing Ni‐supported zeolites derived from agricultural waste, such as rice husks, have exhibited high yields of improved oil. These materials improve mesoporosity for better diffusion, achieving up to 80% oil yield with improved heating values. However, challenges such as catalyst deactivation due to coking and structural collapse remain significant hurdles for long‐term use.

Organometallic catalysts, such as rhodium complexes, have demonstrated exceptional selectivity in the upgrading of bio‐oil through reactions like hydrogenation and carbon–carbon bond formation. These catalysts are particularly valued for their ability to target specific functional groups in complex mixtures, allowing the production of customized hydrocarbon profiles suitable for high‐value fuels such as biojet fuel.^[^ ^107^ ^]^ However, their widespread application is hindered by high costs and limited thermal stability under industrial conditions.

Recent research has addressed these limitations by immobilizing organometallic catalysts onto solid supports such as silica or carbon materials. Immobilization reduces catalyst loss during processing and facilitates recycling and reuse, significantly lowering operational costs.^[^ ^108^ ^]^ Advances in ligand design have also improved the stability and activity of rhodium complexes under harsher conditions, broadening their applicability.^[^ ^107^ ^]^ Furthermore, hybrid approaches that combine organometallic catalysts with other catalytic systems have been explored to synergistically enhance the reaction efficiency while reducing the reliance on costly metals. These efforts are pivotal in transitioning organometallic catalysts from laboratory‐scale applications to more practical, cost‐effective solutions for industrial bio‐oil upgrading processes.

### 7.4 Life Cycle Assessment (LCA)

LCA is a systematic method used to evaluate the environmental impacts associated with all stages of the life of a product, from the extraction of raw materials through production, use and disposal. In the context of bio‐oil upgrading catalysts, LCA considers the environmental footprint of catalyst production (including raw material sourcing and manufacturing), operational performance during bio‐oil upgrading (such as energy use and emissions), and end‐of‐life management (like recycling or disposal). This approach helps identify hotspots where environmental impacts are greatest and supports the development of more sustainable catalysts and processes. By providing a comprehensive view, LCA enables researchers and industry professionals to make informed decisions that balance efficiency, cost, and environmental responsibility throughout the catalyst's entire life cycle.

**Table** **6** shows an example of a life cycle inventory related to 1 MJ of advanced biofuel for the corn stover and lignin‐rich stream scenarios in a study conducted by Zoppi et al.^[^ ^109^ ^]^

**Table 6:** Life cycle inventory of 1 MJ biofuel for Lignin‐Rich stream (LRS) and corn stover (CS) cases.

Inputs/outputs/emissions	Item	LRS‐case	CS case	Unit	Dataset
Inputs	Diesel for harvesting	–	0.023	MJ	Diesel mix at refinery (EU‐28)
	Fertiliser replacement	–	0.90	g N	NPK 15‐15‐15 (nitro phosphate route, 15N‐15P205‐15K20) (EU‐28)
	Biomass transport	–	50	km	Truck, Euro 6, 28–32t gross weight/ 22t payload capacity (GLO)
	Feedstock (wb)	0.27	0.11	kg	–
	Process water	0.017	0.26	kg	Process water (EU‐28)
	Hydrogen	1.2	0	g	Modelled according to^[^ ^129^ ^]^ for electrolysis; Hydrogen steam reforming (DE) for SR
	Electricity	0.095	0.025	kWh_el_	Electricity grid mix (EU‐28)
	Heating	0.020	0.098	kWh_el_	Thermal energy from natural gas (EU‐28)
	Cooling	0.094	0.10	kWh_el_	Modelled according to^[^ ^109^ ^]^
	Platinum (APR catalyst)	0.39	0.65	mg	Modelled according to^[^ ^109^ ^]^
Outputs	Biofuel	23	23	g	–
	Hydrogen	0	0.08	g	–
Emissions	CO_2_	55	38	g	–
	Ash	0.40	1.0	g	Municipal solid waste landfill (EU‐28)
	Wastewater	0.22	0.31	kg	Wastewater treatment (EU‐28)

## 8 Challenges and Future Directions

### 8.1 Catalyst Deactivation and Regeneration

One of the significant challenges in bio‐oil upgrading is catalyst deactivation, which can occur because of various mechanisms. Coking, or the accumulation of carbonaceous deposits on the catalyst surface, is common during processes such as catalytic cracking and hydrodeoxygenation (HDO). These deposits block active sites, reducing catalytic activity and selectivity.^[^ ^110^ ^]^ Poisoning, caused by impurities like sulfur, nitrogen, or metals in bio‐oil, irreversibly damages catalyst surfaces, while sintering the agglomeration of active particles under high‐temperature conditions reduces catalysts’ surface area and activity over time.^[^ ^111^ ^]^

Catalyst longevity is a decisive lever for the techno‐economics of bio‐oil upgrading because the feed is intrinsically heterogeneous and rich in reactive oxygenates, water, and heteroatoms that accelerate deactivation. In hydrodeoxygenation (HDO), the dominant deactivation pathways are 1) coking from condensation/polymerization of phenolics and aldehydes on acid or metal sites, 2) poisoning by nitrogen‐, sulfur‐, chlorine‐, and alkali‐metal species that block active phases (e.g., sulfided NiMo/CoMo, noble metals), 3) hydrothermal sintering of metals and supports driven by high water partial pressure, and 4) phase transformation of sulfided catalysts when sulfur makeup is inadequate. In catalytic cracking/hydrocracking over zeolites or bifunctional catalysts, strong Brønsted acidity promotes desired scission but also accelerates coke growth; repeated coke burn‐off can induce dealumination, loss of acidity, and pore collapse.^[^ ^112^ ^]^ In APR and HTL‐oil hydrotreating, chloride and nitrogen compounds are particularly pernicious; base‐metal catalysts suffer from leaching and sintering in hot condensed phases.

Regeneration strategies are tailored to the active phase. Coked heterogeneous HDO catalysts are commonly regenerated by controlled oxidative burn‐off (air/steam at 450–550 °C), followed by in situ re‐sulfiding (for NiMo/CoMo using DMDS or H_2_S) or reduction (for noble‐metal and carbide/phosphide systems).^[^ ^87^ ^]^ To minimize structural damage, staged temperature ramps and oxygen‐lean atmospheres are used, sometimes preceded by solvent or hydrogen stripping to dislodge weakly bound species. Zeolites undergo periodic air burn‐off of coke; to mitigate gradual acidity loss, operators may re‐acidify (e.g., ammonium exchange) or adopt equilibrium catalyst management (make‐up plus withdrawals).^[^ ^113^ ^]^ To address these issues, research focuses on developing regeneration techniques, such as oxidative treatments to burn off coke or chemical washing to remove poisons. Novel materials such as self‐healing catalysts, which can repair active sites, and structured catalysts with enhanced resistance to deactivation are under development. Improved catalyst formulations, including bimetallic or doped materials, also aim to extend the catalyst lifetimes by minimizing these degradation pathways.

The challenges associated with bio‐oil upgrading are multifaceted, as shown in **Table** **7**. Catalyst deactivation, often due to coking or sintering, remains a central issue that can be mitigated through improved catalyst design and regular regeneration.^[^ ^114^ ^]^ Hydrogen availability is another major bottleneck; considering its cost and logistic limitations, on‐site or renewable hydrogen production methods are gaining traction.^[^ ^115^ ^]^ The high‐water content in raw bio‐oil impacts both fuel quality and process stability, necessitating either physical separation techniques or the use of water‐tolerant catalysts. Inadequate selectivity often leads to inefficient conversions, which can be addressed using customized catalysts with controlled acidity or dual functionality. Finally, the thermal instability of bio‐oil requires a careful balance in the processing conditions to avoid degradation while still achieving efficient upgrading.^[^ ^116^ ^]^ Together, these strategies aim to improve the feasibility and scalability of sustainable biofuel production.

**Table 7:** Outlining the key limitations in bio‐oil upgrading processes and the proposed solutions.

Limitation	Description	Proposed Solutions
Catalyst deactivation	Due to coking, sintering, or poisoning by impurities	Use coke‐resistant materials, apply regeneration cycles, or develop sintering‐resistant catalysts
Hydrogen supply issues	High cost and limited availability of hydrogen	Integrate on‐site hydrogen production (e.g., reforming), explore bio‐derived hydrogen sources
High water content in bio‐oil	Lowers energy density and catalyst stability	Pretreatment of feedstock, phase separation, or water‐tolerant catalysts
Poor selectivity	Unwanted side products due to broad reaction pathways	Tailor acidity/basicity, employ bifunctional or shape‐selective catalysts
Thermal instability of bio‐oil	Degradation or polymerization at high temperatures	Optimize reaction conditions, apply mild hydroprocessing steps

### 8.2 Scale‐Up and Commercialization

Scaling catalytic bio‐oil upgrading processes to industrial levels poses several challenges. The heterogeneity of biomass feedstocks and the complexity of bio‐oil compositions require flexible and robust catalysts capable of handling diverse conditions. Moreover, the high costs of hydrogen, essential for processes such as HDO and energy‐intensive operations, can hinder the economic feasibility. Integrating catalytic processes with existing refining infrastructure while maintaining competitiveness with fossil fuels is another critical hurdle.^[^ ^117^ ^]^

Advances in pilot‐scale and commercial operations are promising. Facilities employing technologies such as HTL and pyrolysis coupled with upgrading are being tested but face challenges in ensuring process consistency and efficiency. Collaborations between academic institutions, industries, and governments are crucial to addressing these barriers. Additionally, technoeconomic analyses and LCAs guide optimization efforts to make these processes sustainable and cost‐effective.

### 8.3 Toward 100% Sustainable Biojet Fuel

Achieving fully sustainable biojet fuel requires technological, regulatory, and economic challenges. From a technical perspective, the integration of renewable hydrogen production, such as through water electrolysis powered by renewable energy, is crucial to reducing the carbon footprint of HDO processes. Moreover, efforts to use circular bioeconomy principles, such as recycling catalysts and valorizing byproducts such as biochar, are vital to achieving sustainability.^[^ ^118^ ^]^

Regulatory support through subsidies, carbon credits, and mandates for SAF blends is essential to stimulate industry investment. Partnerships between governments, aviation industries, and renewable energy sectors can accelerate the deployment of biojet fuel technologies.^[^ ^119^ ^]^

Future opportunities lie in coupling bio‐oil upgrading with other green technologies, such as direct air capture of CO_2_ or biogas upgrading, to enhance the overall sustainability of the fuel production chain. The emphasis on developing efficient logistics for biomass collection and conversion is equally critical. Together, these advances pave the way for biojet fuel to play a central role in decarbonizing the aviation sector.

## 9 Conclusion

### 9.1 Summary of Key Findings

This review highlights significant advances in the field of bio‐oil upgrading for sustainable biojet fuel production. Biomass‐derived bio‐oil, produced through thermochemical methods such as pyrolysis and HTL, holds immense potential as a renewable feedstock for aviation fuels. However, its high oxygen content, instability, and diverse composition challenges require sophisticated upgrading technologies. Catalytic processes such as hydrodeoxygenation (HDO), catalytic cracking, and hydrocracking have emerged as effective pathways for the transformation of bio‐oil into hydrocarbon fuels. Key innovations in catalyst design, including bimetallic systems, hierarchical zeolites, and carbon‐supported materials, have significantly enhanced these processes’ efficiency, selectivity, and sustainability.

Despite these advances, challenges remain in scaling these technologies, mitigating catalyst deactivation, and integrating processes into existing industrial infrastructures. Moreover, case studies have demonstrated the viability of various catalytic systems but emphasize the need for further optimization to address economic and operational limitations.

### 9.2 Implications for the Field of Biojet Fuel Production

The progress outlined in this review underscores the pivotal role of catalysis in the advancement of biojet fuel production. Catalysts enable the efficient removal of oxygen and the selective conversion of bio‐oil into hydrocarbons compatible with aviation fuel standards. The integration of novel catalytic materials and reaction conditions into pilot‐scale facilities demonstrates the feasibility of these technologies for commercial applications. Furthermore, the focus on sustainability, such as the use of renewable hydrogen and the principles of circular bioeconomy, aligns with global goals for decarbonizing the aviation sector.

However, the success of biojet fuel as a mainstream alternative depends not only on technological advancements but also on supportive policies and industrial partnerships. The aviation industry requires clear regulatory frameworks and incentives to adopt sustainable aviation fuels at scale. Furthermore, continued investment in research and development is essential to overcome economic and logistical barriers, ensuring that biojet fuel becomes a viable component of the global energy transition.

### 9.3 Future Outlook on Research and Commercial Application of Biojet Fuel

Although significant progress has been made in bio‐oil upgrading for sustainable biojet fuel production, further research is essential to overcome the remaining challenges. A key area of focus is catalyst innovation, where efforts should aim at developing materials with enhanced resistance to deactivation, higher selectivity, and lower costs. Promising advances include self‐regenerating and recyclable catalysts that can sustain performance over extended operational periods. Additionally, process optimization is crucial to refine reaction conditions, ensuring maximum yields while minimizing energy consumption and operational costs.

Integration with renewable technologies offers another exciting avenue, particularly the coupling of bio‐oil upgrading with renewable hydrogen production, carbon capture, and waste valorization systems, which can contribute to fully sustainable fuel production chains. Addressing scaling and economic viability challenges requires comprehensive technoeconomic analyses to guide the transition of processes from laboratory research to commercial‐scale operations. Finally, establishing supportive policy frameworks and fostering collaboration between academic institutions, industries, and governments are essential to incentivize the production, adoption, and investment in biojet fuels.

In conclusion, the journey toward SAFs derived from biomass is complex and promising. By leveraging technological advancements and fostering cross‐sector collaboration, biojet fuel has the potential to significantly contribute to global energy sustainability and reduce the aviation industry's carbon footprint.

## Conflict of Interest

The authors declare no conflict of interest.

## Author Contributions

**Thandiswa Jideani**: writing—review and editing, writing—original draft, methodology, investigation, formal analysis, conceptualization. **Ntalane Sello Seroka**: writing—review and editing, visualization, validation, supervision, methodology, formal analysis, conceptualization. **Lindiwe Khotseng**: writing—review and editing, validation, supervision, resources, project administration, funding acquisition, conceptualization.
